# Clinical dose effect and functional consequences of R92Q in two families presenting with a TRAPS/PFAPA‐like phenotype

**DOI:** 10.1002/mgg3.229

**Published:** 2017-01-14

**Authors:** Sylvie Grandemange, Sébastien Cabasson, Guillaume Sarrabay, Jérôme Pène, Cécile Rittore, Elodie Sanchez, Marie‐Caroline Chastang, Gaël Guyon, Pascal Pillet, Isabelle Touitou

**Affiliations:** ^1^ Département de génétique médicale, maladies rares et médecine personnaliséeCHRU de MontpellierMontpellierFrance; ^2^ INSERM UMR1183IRMBMontpellierFrance; ^3^ Service de pédiatrieCHU de PauPauFrance; ^4^ Centre de référence des maladies autoinflammatoiresCeRéMAICHRU de MontpellierMontpellierFrance; ^5^ Université de MontpellierMontpellierFrance; ^6^ Service de pédiatrie médicaleCHRU de BordeauxBordeauxFrance; ^7^ Service de pédiatrie généraleCHRU de MontpellierMontpellierFrance

**Keywords:** Clinical dose effect, R92Q, *TNFRSF1A*, TRAPS recessive

## Abstract

**Background:**

TNF receptor‐associated syndrome (TRAPS) is a dominantly inherited autoinflammatory condition caused by mutations in the *TNFRSF1A* gene. The mechanism underlying the variable expressivity of the common variant R92Q (rs4149584; c.362G>A; p.Arg121Gln) is unclear and is of critical importance for patient care and genetic counseling. This study evaluated the impact of the number of R92Q mutations in two unique unrelated families.

**Methods:**

Two patients with undefined but clear autoinflammatory symptoms were referred for genetic diagnosis. Blood samples were collected from the available family members to screen autoinflammatory genes and assess key steps of the TNFR1‐mediated signaling pathway using flow cytometry and ex vivo culture.

**Results:**

R92Q homozygosity was demonstrated for the two probands. In family 1, the segregation analysis revealed TRAPS‐like symptoms in all carriers, with a more severe presentation in the proband, whereas in family 2, the heterozygous parents were totally asymptomatic, suggesting recessive transmission. Functional studies revealed a nonclassical pathogenesis of TRAPS in the two probands and suggested a compensatory mechanism without clear dose effect.

**Conclusion:**

We observed for the first time a possible clinical dose effect of R92Q. This work highlights the importance of familial studies to reconcile the contradictory reports published on the pathogenicity of this variant.

## Introduction

Systemic autoinflammatory disorders (SAIDs) are rare disorders of the innate immunity. Among them, TNF receptor‐associated periodic syndrome (TRAPS; OMIM #142680), a classically dominant recurrent fever, is caused by mutations in the *TNFRSF1A* gene (OMIM #191190) (McDermott et al. 1999). TRAPS is mainly characterized by prolonged recurring inflammatory attacks of fever, abdominal pain, arthritis, and cutaneous manifestations. Genetic and phenotypic heterogeneity are common in TRAPS (Lachmann et al. 2014) and the mechanisms by which heterozygous mutations cause TRAPS seem complex and probably cell‐ and variant‐dependent (Rigante et al. 2014). A spectrum of genetic variants with various pathogenic effects has been recorded (infevers http://fmf.igh.cnrs.fr/ISSAID/infevers/). The clinical significance of one of them, historically called R92Q (rs4149584, c.362G>A; p.Arg121Gln, NP_001056.1), is most debated. Whether R92Q is a true mutation, a low‐penetrance variant, a functional polymorphism, or a susceptibility factor for inflammation is elusive. Indeed, its frequency in the general population is quite elevated (>1%), most R92Q carriers exhibit mild TRAPS symptoms, and functional studies demonstrated intermediate behaviors. Moreover, association of R92Q with multifactorial diseases has been reproducibly demonstrated (Touitou 2013). Therefore, the role of R92Q in the patient phenotype remains obscure and only a handful of isolated homozygous individuals with autoinflammatory symptoms have been described (Ravet et al. 2006; Pelagatti et al. 2011; Celebi‐Tayfur et al. 2013; Abdwani et al. 2015). Here, we provide the first familial investigation of the impact of the number of R92Q variants at both clinical and functional levels.

## Materials and Methods

### Ethical compliance

All patients, their family members, and the healthy donors gave informed consent for their inclusion in this study. The collection of samples is declared for research (number DC‐2012‐1579). Blood samples from controls were recruited from French blood agency (EFS; Convention EFS‐PM n° 21PLER2015‐0013).

### Healthy controls and patients samples

Medical charts were completed and updated through a telephonic contact to disclose any recent changes in their clinical status. The healthy donors were adults like the parents, so as to limit the risk of late occurrence of symptoms. They were sex‐matched with the patients.

Blood samples from healthy donors and the members of the two families were collected in heparin tubes. In all samples, the CRP levels quantified by ELISA were below the normal limit (<10 mg/L), indicating no obvious inflammatory manifestation at the time of the sampling (Table S1). Only the two probands had cortisone treatment at the time of blood collection.

Cells were separated from plasma by cold centrifugation for 5 min at 300 g. Plasma samples were stored at −80°C until analysis and peripheral blood leukocytes (PBLs) were purified by removing erythrocytes with a blood lysis buffer containing 155 mmol/L NH4Cl, 10 mmol/L KHCO3, and 1 mmol/L EDTA. Cells were stored in cold cell culture freezing medium (Gibco^®^) in liquid nitrogen until analysis.

### Genetic diagnosis

Genomic DNA was extracted from circulating white blood cells, then *TNFRSF1A* (OMIM **#**191190, NM_001065.3, all exons), *MEFV* (OMIM **#**608107**,** NM_000243.2, exons 2‐10), NLRP3 (OMIM **#**606416, NM_001243133.1, exon 3), and MVK (OMIM **#**251170, NM_000431.2, all coding exons) were Sanger‐sequenced according to conventional methods. Next‐generation sequencing (NGS) encompassing a panel of 32 validated and candidate autoinflammatory genes (list available upon request) was also undertaken for the two probands to detect possible pathogenic mutations in other SAID genes and/or oligogenism. The exons of the genes were captured using a Nextera Rapid Capture Custom kit (Illumina), sequenced on a MiSeq (Illumina, France) equipment, and reads were interpreted using the SeqNext software (JSI medical system, France).

### Functional studies

A detailed description of functional studies can be found in supporting information.

The membrane and intracellular expression of TNFR1 was studied by flow cytometry using a FITC‐conjugated anti‐TNFR1 monoclonal antibody (mAb) and an APC‐conjugated anti‐CD14 mAb to identify monocytes. Data acquisition was done using a FACSCalibur flow cytometer and fluorescence was analyzed using the CellQuest Pro software.

To evaluate the TNF‐induced cytokine productions, peripheral blood leukocytes (PBLs) were incubated in Yssel's culture medium with complete medium alone or with TNF (100 ng/mL) for 6 h (Pène et al. 2008). After incubation, cell‐free supernatants were stored at −80°C until analysis. The soluble form of TNFR1 (sTNFR1), cytokines concentrations (IL‐6, IL‐8, MCP‐1, and IL12‐p70) in plasma, and cell culture supernatants were measured using commercial kits according to manufacturer's instructions.

## Results

### Case reports

**Figure 1:**
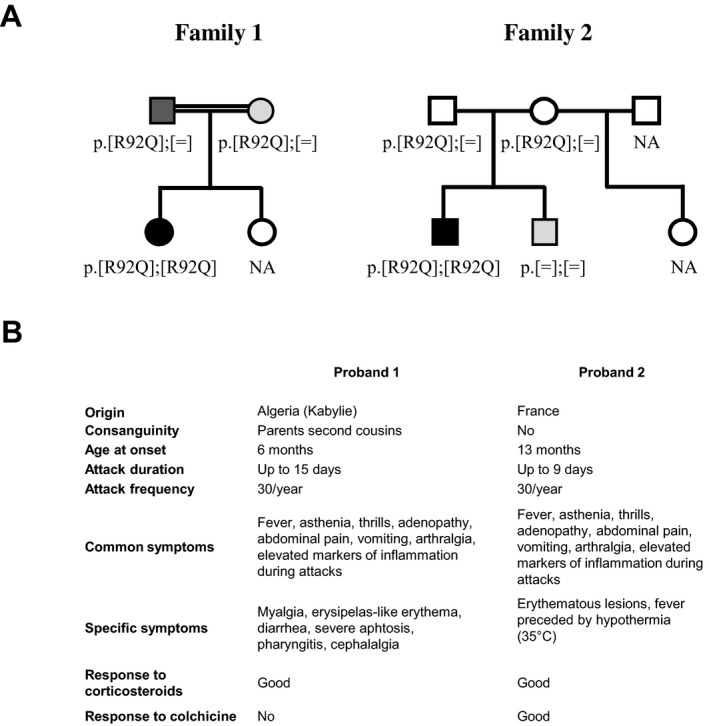
The family structure and the main clinical features of the two probands (A) R92Q segregation in two families. Members of two unrelated families, and their respective genotypes at the *TNFRSF1A* gene (OMIM #191190; NM_001065.3), are represented. Black, dark gray, light gray, and white symbols represent individuals with a clear autoinflammatory picture (i.e., probands), patients with mild symptoms, patients with evanescent symptomatology in the early childhood, and totally asymptomatic individuals, respectively. The parents of patient 1 were second degree cousins (double line). (B) Comparative epidemiological and clinical data for the two probands.

The family structure and the main clinical features of the two probands are summarized in Fig. 1. Common characteristics were recurrent fever, asthenia, thrills, adenopathy, abdominal pain, vomiting, and arthralgia with marked biological inflammation (elevated neutrophils, high levels of C‐reactive protein, and erythrocyte sedimentation rate). These symptoms were absent between the flares.

#### Family 1

The proband was referred at age 14 years for genetic characterization of her recurrent fevers. Periodic fever, aphthous stomatitis, pharyngitis, cervical adenitis syndrome (PFAPA) was the first diagnostic envisaged. Her Algerian origin, consanguinity and clinical features such as, abdominal pain, arthralgia, and erysipelas‐like erythema were in favor of familial Mediterranean fever (FMF: OMIM #249100). Other evocated SAIDs were TRAPS (long‐lasting attacks, myalgia), mevalonate kinase deficiency (OMIM #260920) and Behçet's disease (OMIM #109650). She had severe ulcerations of tongue, anus and vagina, but periorbital edema, uveitis, immunizations as a triggering factor, and mevalonic aciduria during flares were absent. HLA‐B51 and B27 were negative.

Non‐steroidal anti‐inflammatory therapeutics were ineffective. Corticosteroids (Prednisone, 40 mg/day) were necessary for several days and significantly reduced attack duration. Colchicine was tried but quickly appeared ineffective and badly tolerated. Anti‐IL‐1 treatment was refuted by the family. Clinical examination at age 17 years revealed persistent fever, lasting up to 5 or 6 days, twice a month, only attenuated by corticosteroids. Her quality of life was altered with frequent absences from school, sadness, and low self‐esteem. Genetic analysis of the *TNFRSF1A* gene disclosed R92Q apparent homozygosity and no mutation in the other autoinflammatory genes investigated.

Both parents were carriers of a single R92Q allele confirming biallelic R92Q in the proband. Milder symptoms were highlighted in the parents upon in‐depth interrogation. The mother remembered episodes of unexplained fever during her childhood that spontaneously stopped in adulthood. The father had more severe symptoms with aphthous stomatitis and fever that are still present, but with a lower frequency and a globally little impact on his quality of life. To evaluate the possibility that another SAID gene was responsible for the disease in this family, NGS was undertaken and did not reveal homozygous mutation or clearly heterozygous pathogenic mutation segregating with the phenotype. The proband's sister was asymptomatic and unavailable for genetic analysis. A clinical dose effect was therefore observed in this family.

#### Family 2

The proband of this family also suffered from recurrent fever since the age of 13 months. Fever could rise up to 42°C, lasted 8–9 days, and was preceded by marked hypothermia (35°C). At the age of 3 years, symptoms became even more pronounced with abdominal pain, vomiting, arthralgia, and erythematous lesions on ankles and elbows. Corticosteroids were promptly tried, and 2 mg/kg of prednisone during 4 days were mandatory to stop fever. Colchicine (1/2 mg/day), given to reduce prednisone intake, dramatically reduced the number and intensity of the fever episodes. At last visit (age 6 years), growth was within the normal range and the patient could live normally, except that he manifested an exacerbated sensitivity to staphylococcal infections. *TNFRSF1A* screening revealed R92Q homozygosity. The parents were French and unrelated. They were both heterozygous for R92Q and totally asymptomatic. As the proband's brother experienced abdominal pain and fever during infancy and although they spontaneously disappeared, we checked *TNFRSF1A* but no mutation was detected. To evaluate the possibility that another SAID gene was responsible for the disease in this family, NGS was undertaken and only disclosed p.Ala755Val (rs61747625; c.2264C>T; NP_071445.1) in *NOD2* (NM_022162.1; OMIM #605956), a Crohn's‐associated polymorphism in the proband, father, and the brother (OMIM #266600) (Lesage et al. 2002). The step‐sister was asymptomatic and unavailable for genetic analysis.

### Functional studies

We collected blood samples from the two homozygous probands and their available relatives, and from healthy donors (*n* = 13) not carrying the R92Q variant. All results were compared according to the number of the R92Q alleles.

#### Systemic levels of inflammatory biomarkers

**Figure 2:**
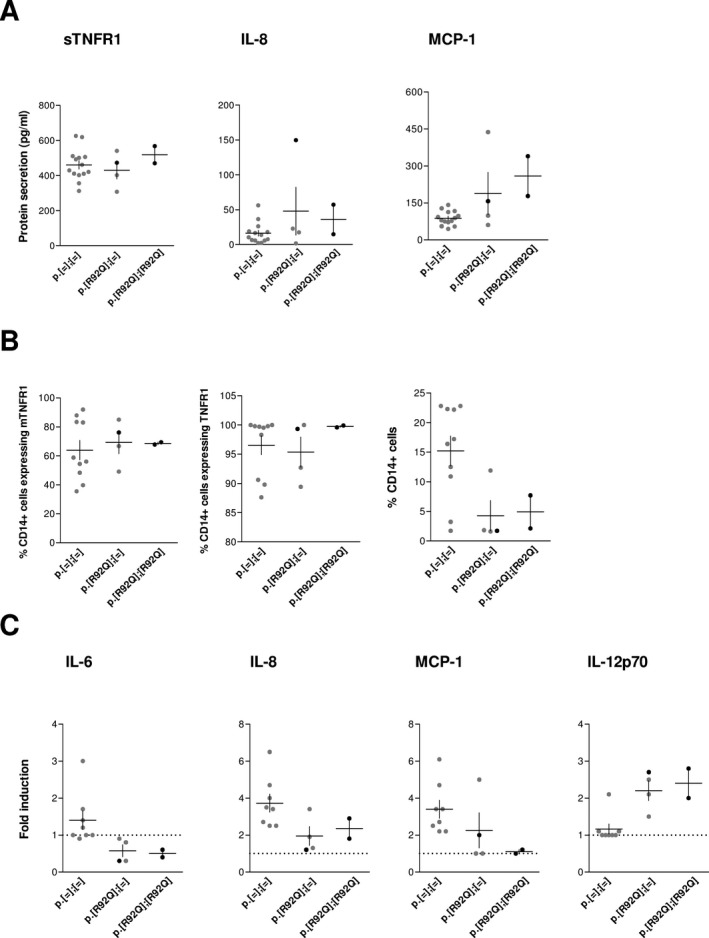
Functional effect of the number of R92Q variants on inflammatory responses. (A) Inflammatory biomarkers in plasma samples. The concentration of sTNFR1, IL‐8, and MCP‐1 was measured by ELISAs. The asymptomatic and symptomatic individuals in each group are denoted by gray and black dots, respectively. The levels were compared according to the number of R92Q variants. The mean and SEM of each genotype group is represented by horizontal and vertical lines, respectively. (B) TNFR1 expression in monocytes. The CD14 +  cells expressing the membrane (mTNFR1, left) and intracellular (middle) TNFR1 and the relative percentage of CD14 +  cells (right) are quantified by flow cytometry in PBLs. The data are represented as indicated in A. (C) Ex vivo cytokine production in response to TNF. PBLs were incubated in complete medium alone or with TNF. After incubation, the cytokine concentrations were quantified by ELISAs. The fold induction of each cytokine relative to spontaneous production in the absence of TNF is shown. Data were analyzed and denoted as in A. The horizontal dot line denotes a fold induction of one, which is indicative of no variation in cytokine production.

Soluble TNFR1 (sTNFR1) and IL‐8 plasma levels did not differ between the three groups (Fig. 2A), whereas IL‐6 and TNF were undetectable (Table S1). However, we observed a tendency to increased systemic MCP‐1 levels in the heterozygous and homozygous R92Q carriers with a slight dose effect.

#### Membrane and intracellular TNFR1 expression

We examined the TNFR1 trafficking in blood monocytes as it is often impaired in TRAPS patients (Rigante et al. 2014). Flow cytometry showed that the percentages of CD14 +  monocytes expressing the membrane and intracellular forms of TNFR1 were comparable between the three groups (Fig. 2B). The median fluorescence intensity did not vary either (Fig. S1), suggesting that the presence of R92Q did not result in membrane expression defect and/or intracellular accumulation of TNFR1. However, we observed a decreased percentage of CD14 +  cells in individuals with one or two R92Q variants. This result was replicated on a second sample from three of the healthy donors, proband 2, and his parents, supporting that this decrease was not due to sample collection procedures.

#### TNF‐induced cytokine productions

We also investigated the TNF‐induced cytokine productions in PBLs ex vivo cultures. The spontaneous releases of sTNFR1 and a panel of cytokines (Supporting information) were similar in the three groups (data not shown). After TNF stimulation, we detected no (IL‐6), or less (IL‐8 and MCP‐1) cytokine induction in individuals with one or two R92Q variants as compared to the noncarriers, with a trend toward a dose effect for MCP‐1 secretion (Fig. 2C). No cytokine induction was detected after LPS stimulation either (data not shown), suggesting that this induction defect was not specific to TNF‐mediated signaling. Surprisingly, we observed an apparent induction of IL‐12p70 in response to TNF in the R92Q‐expressing cells only (Fig. 2C). ROS production by nonstimulated and TNF‐stimulated PBLs and cytotoxicity were comparable between the three groups (Fig. S2), indicating no particular susceptibility to stress or death of the cells expressing the R92Q variant.

## Discussion

As the spectrum of SAIDs is exponentially expanding over years, genetic and differential diagnosis remains challenging. Interpretation of the clinical significance of R92Q in *TNFRSF1A* is an example of such an issue. R92Q is a variant associated with a mild TRAPS phenotype that moderately affects TNFR1 trafficking. In contrast, severe phenotype is observed in patients carrying structural cysteine mutations, which have a clear impact on subcellular localization. These dual effects strongly suggest a different pathogenesis leading to the constant hyperinflammation observed with R92Q.

In the two families reported here, individuals with one R92Q variant had no or less symptoms as compared to the two homozygous probands who both had quite severe and similar phenotype. These results suggest a dose effect of the R92Q variant at the clinical level in family 1, and a possible recessive transmission in family 2. The latter echoes a recent study also suggesting recessive TRAPS in a family with this variant (Abdwani et al. 2015). Cooperation between TNFR1 structural mutations and the wild‐type protein results in dominant TRAPS (Simon et al. 2010). Our results suggest that another molecular mechanism, possibly interaction with different genetic factors, underlies the phenotype observed in R92Q homozygotes (Touitou 2013).

We assessed for the first time the possible functional consequences of the number of R92Q variants on TNFR1‐mediated signaling involved in TRAPS physiopathology. The apparent dose effect of the R92Q variant at the clinical level was not detected at any step of this classical TRAPS signaling pathway. Indeed, the known associated defects, that is, hyperexpression of inflammatory mediators, impaired receptor trafficking, and high ROS production (Rigante et al. 2014), were not observed, whatever the number of R92Q alleles.

Instead, we show here the increased MCP‐1 plasmatic levels and TNF‐induced IL‐12p70 release that might reflect a compensatory downstream signaling resulting in the inflammatory phenotype observed in the two probands and some of the R92Q carriers. A compensatory mechanism involving MCP‐1 was previously suggested for the TRAPS patients carrying the R92Q variant (Rebelo et al. 2009). This hypothesis was consistent with the altered conformation of R92Q‐TNFR1, which can confer distinct and specific intracellular consequence (Lewis et al. 2012). However, in contrast to previous studies (Nedjai et al. 2008; Rebelo et al. 2009; Simon et al. 2010; Greco et al. 2015), diminished rather than augmented response to TNF was detected in our R92Q patients. As the membrane and intracellular expression of TNFR1 were not modified in the R92Q carrying group, the lack of response to TNF might be linked to a defective downstream signaling of the TNFR1 receptor, and/or to a decrease in the CD14 + ‐expressing cells which are both supported by our results. Further experiments, including more families and/or investigating alternative inflammatory processes, could help relate this compensatory mechanism to the dose effect of the R92Q variant observed at the clinical level.

In conclusion, our study revealed a new, nonclassical pathogenesis of TRAPS associated with R92Q homozygosity, in two families presenting with a TRAPS/PFAPA‐like phenotype. We hypothesize the interaction of the R92Q variant with other genetic factors leading to inflammatory phenotype. Altogether, our findings emphasize the importance of combining information from segregation analysis, extended sequencing strategy, and where possible functional studies to evaluate variant pathogenicity and provide appropriate management and genetic counseling to the patients.

## Conflict of Interest

The authors declare no competing financial interests.

## Supporting information


**Data S1.** Materials and methods.
**Table S1**. CRP, sTNFR1, and cytokine concentrations in plasma of healthy donors and patients.
**Figure S1.** Membrane and intracellular TNFR1 protein expression in monocytes.
**Figure S2.** ROS production and cytotoxic assay.
