# Supplementary material for: Clinical dose effect and functional consequences of R92Q in two families presenting with a TRAPS/PFAPA‐like phenotype
Source: Mol Genet Genomic Med. 2017 Jan 14;5(2):110–6. doi: 10.1002/mgg3.229 (PMC5370222; doi:10.1002/mgg3.229)
Supplement: Supplementary file 1 — Data S1. Materials and methods. Table S1. CRP, sTNFR1, and cytokine concentrations in plasma of healthy donors and patients. Figure S1. Membrane and intracellular TNFR1 protein expression in monocytes. Figure S2. ROS production and cytotoxic assay. [file MGG3-5-110-s001.docx]

**Supporting information**

**Clinical dose effect and functional consequences of R92Q in two families presenting with a TRAPS/PFAPA-like phenotype**

Sylvie Grandemange^1,2*^, Sébastien Cabasson^3*^, Guillaume Sarrabay^1,2,4,5^, Jérôme Pène^2^, Cécile Rittore^1,2^, Elodie Sanchez^1,2^ Marie-Caroline Chastang^6^, Gaël Guyon^7^, Pascal Pillet^6^, Isabelle Touitou^1,2,4,5^

1. Département de génétique médicale, maladies rares et médecine personnalisée, CHRU de Montpellier, France
2. INSERM UMR1183, IRMB, Montpellier, France
3. Service de pédiatrie, CHU de Pau, France
4. Centre de référence des maladies autoinflammatoires, CeRéMAI, CHRU de Montpellier, France
5. Université de Montpellier, France
6. Service de pédiatrie médicale, CHRU de Bordeaux, France
7. Service de pédiatrie générale, CHRU de Montpellier, France

^*^These authors equally contributed to this work

**MATERIALS AND METHODS**

**Expression of TNFR1**

The membrane and intracellular expression of TNFR1 was studied by flow cytometry. After thawing, 2x10^5^ PBLs were stained with either a FITC-conjugated anti-TNFR1 monoclonal antibody (mAb) (Santa Cruz) or a matching isotype control (BD Biosciences). To identify monocytes, double stainings were carried out by simultaneous incubation with an APC-labeled mAb recognizing the surface markers CD14 (BD Biosciences). Each mAb was used at the concentration recommended by the manufacturers and stainings were performed at 4°C in the dark. For analysis of intracellular TNFR1 expression, cells were first stained with anti-CD14-APC mAb as mentioned above. After fixation and permeabilization with Fixation/Permeabilization buffer (BD Biosciences) according to the manufacturer’s instructions, cells were stained with the FITC-conjugated anti-TNFR1 mAb, and finally washed with the Perm/Wash™ buffer (BD Biosciences) before being analyzed for fluorescence. Data acquisition was done by using a FACSCalibur flow cytometer and fluorescence analyzed using the CellQuest Pro software (both from BD Biosciences).

**TNF stimulation and ROS detection**

PBLs were seeded at 1x10^6^ cells/ml in Yssel’s culture medium supplemented with 1% heat-inactivated AB+ human serum (Etablissement Français du Sang, Toulouse, France) as previously described. Cells were cultured in 24-well plates and incubated with complete medium alone or with TNF (100 ng/ml) for 6 h at 37°C in a humidified atmosphere containing 5% CO2. Each sample and culture condition were assayed at least in duplicates. After incubation, adherent cells and cells in suspension were harvested and centrifuged. Cell free supernatants were stored at -80°C until analysis. Pelleted cells were resuspended in Hank's Balanced Salt Solution (HBSS, Gibco^®^) containing 10 µM of di(acetoxymethyl ester) 6-carboxy-2′,7′-dichlorodihydrofluorescein diacetate, (AM-H_2_DCFDA, Molecular Probes) and incubated 30 min at 37°C in the dark. As an indicator for reactive oxygen species (ROS) in cells, the green-fluorescent form of AM-H_2_DCFDA was measured using a fluorescence plate reader (Varioskan Flash, ThermoScientific) with excitation at 493 nm and emission at 527 nm.

**CRP, sTNFR1, and cytokines quantifications**

C-reactive protein (CRP) was quantified in plasma samples by ELISA (Quantikine® Elisa, Bio-Techne – R&D Systems). The soluble form of TNFR1 (sTNFR1), cytokines and chemokines concentrations in plasma and cell culture supernatants were measured using commercial DuoSet ELISA kits from R&D Systems (sTNFR1, IL-1β, IL-1α, IL-33, IL-17, IL-22) or kits from e-biosciences (TNF-α, IL-6, IL-8, IL-18, IL-10, IFN-α, MCP-1, IL-12p70) according to manufacturer’s instructions. IL-4 and IFN-γ cytokines were measured as previously described (1). Concentrations below the detection limit were considered as not detectable (ND).

**Cytotoxic assay**

The cellular cytotoxicity was quantified by colorimetric method using the Pierce^TM^ LDH Cytotoxicity Assay Kit (Pierce) in cell culture supernatants.

| **Supplementary Table S1** CRP, sTNFR1 and cytokine concentrations in plasma of healthy | | | | | | | | | | | | | | | | | |
| --- | --- | --- | --- | --- | --- | --- | --- | --- | --- | --- | --- | --- | --- | --- | --- | --- | --- |
| donors and patients | | |  |  |  |  |  |  |  |  |  |  |  |  |  |  |  |
|  |  |  |  |  |  |  |  |  |  |  |  |  |  |  |  |  |  |
|  | **TNFR1 variant** | p.[=];[=] | | | |  | p.[R92Q];[=] | | | | | |  | p.[R92Q];[R92Q] | | | |
|  | **Phenotype** | Asymptomatic | | | |  | Asymptomatic | | | | | Symptomatic |  | Symptomatic | | | |
|  | **n =** | 14 | | | |  | 3 | | | |  | 1 |  | 2 | | | |
|  |  |  |  |  |  |  |  |  |  |  |  |  |  |  |  |  |  |
|  | **CRP** | 1,3 | ( | 1,5 | ) |  | 0,8 | ( | 0,1 | ) |  | 2,4 |  | 0,6 | ( | 0,2 | ) |
|  | **sTNFR1** | 460,9 | ( | 89,2 | ) |  | 416,5 | ( | 116,9 | ) |  | 472,1 |  | 518,8 | ( | 69,2 | ) |
|  | **IL-1β** | 5,7 | ( | 4,5 | ) |  | 11,2 | ( | 12,5 | ) |  | ND |  | 6,9 | ( | 4,1 | ) |
|  | **IL-1α** | ND |  |  |  |  | ND |  |  |  |  | ND |  | ND |  |  |  |
|  | **IL-18** | 597,4 | ( | 330,8 | ) |  | 205,4 | ( | 101,5 | ) |  | 482,0 |  | 191,1 | ( | 76,1 | ) |
|  | **IL-33** | 179,2 | ( | 228,1 | ) |  | 71,7 | ( | 89,6 | ) |  | ND |  | ND |  |  |  |
|  | **TNF** | 6,1 | ( | 7,8 | ) |  | 5,0 | ( | 1,7 | ) |  | ND |  | ND |  |  |  |
|  | **IL-6** | ND |  |  |  |  | 5,2 | ( | 5,5 | ) |  | 3,4 |  | ND |  |  |  |
|  | **IL-12p70** | 11,9 | ( | 16,1 | ) |  | 68,5 | ( | 106,6 | ) |  | ND |  | 34,8 | ( | 39,2 | ) |
|  | **IFNα** | 17,5 | ( | 39,3 | ) |  | 23,5 | ( | 28,6 | ) |  | ND |  | 113,5 | ( | 150,5 | ) |
|  | **IL-8** | 16,2 | ( | 15,0 | ) |  | 14,0 | ( | 11,0 | ) |  | 149,6 |  | 36,0 | ( | 29,9 | ) |
|  | **MCP-1** | 87,9 | ( | 28,8 | ) |  | 198,6 | ( | 207,9 | ) |  | 156,9 |  | 258,8 | ( | 114,4 | ) |
|  | **IL-17** | 43,8 | ( | 80,0 | ) |  | 329,1 | ( | 272,8 | ) |  | ND |  | ND |  |  |  |
|  | **IL-22** | ND |  |  |  |  | ND |  |  |  |  | ND |  | ND |  |  |  |
|  | **IL-4** | ND |  |  |  |  | ND |  |  |  |  | ND |  | ND |  |  |  |
|  | **IL-10** | ND |  |  |  |  | 7,5 | ( | 9,5 | ) |  | ND |  | ND |  |  |  |
|  | **IFNγ** | 74,0 | ( | 119,6 | ) |  | 26,2 | ( | 29,9 | ) |  | ND |  | 125,8 | ( | 165,1 | ) |
|  |  |  |  |  |  |  |  |  |  |  |  |  |  |  |  |  |  |
| Mean (SD) of plasma CRP (mg/l), sTNFR1 (pg/ml) and cytokine concentrations (pg/ml) | | | | | | | | | | | | | | | | |  |
| Concentrations below the detection limit of ELISAs were considered as not detectable (ND) | | | | | | | | | | | | | | | | |  |

**SUPPLEMENTARY FIGURES**

**Supp. Fig. S1 : Membrane and intracellular TNFR1 protein expression in monocytes.** Peripheral blood leukocytes (PBLs) were prepared and stained as indicated in supplemented Materials and Methods. Each dot represents the median fluorescence intensity (MFI) of the membrane (A) and intracellular (B) TNFR1 expression in monocytes for each individual. Data were analyzed and compared as indicated in the legend of Figure 2. For the proband and his heterozygous parents of the family 2, and three healthy donors, two sample collections were obtained and the mean of the two experiments is represented.

**Fig. S2 : ROS production and cytotoxic assay.**

PBLs from healthy donors (n=8) and the members of the two families (the two homozygous probands and their parents, n=6) were seeded at 1x10^6^ cells/ml in Yssel’s culture medium supplemented with 1% heat-inactivated AB+ human serum. Cells were cultured in 24-well plates and incubated with complete medium alone or with TNF (100 ng/ml) for 6 h, as exposed in the legend of Figure 2.

**A-** After incubation, adherent cells and cells in suspension were harvested and centrifuged. Pelleted cells were resuspended in Hank's Balanced Salt Solution (HBSS, Gibco) containing 10 µM of AM-H2DCFDA and incubated 30 min at 37°C in the dark. As an indicator for total reactive oxygen species (ROS) in cells, the green-fluorescent form of AM-H2DCFDA was measured using a fluorescence plate reader with excitation at 493 nm and emission at 527 nm. The results are analyzed and presented as reported in the legend of Figure 2. The mean of the values obtained in technical duplicate or triplicate for each sample has been plotted as a dot and each dot represents the spontaneous production of ROS in untreated culture condition (left) or the fold induction of ROS in response to TNF stimulation as compared to untreated condition (right). The horizontal dot line is indicative of no ROS production variation.

**B-** The cellular cytotoxicity was quantified by colorimetric method using the PierceTM LDH Cytotoxicity Assay Kit (Pierce) in the cell culture supernatants and results are represented as in A.

**Reference**

1. Pène J, Chevalier S, Preisser L et al. Chronically inflamed human tissues are infiltrated by highly differentiated Th17 lymphocytes. J Immunol 2008: 180: 7423-7430.
